# A Bio‐Inspired Perspective on Materials Sustainability

**DOI:** 10.1002/adma.202413096

**Published:** 2025-01-05

**Authors:** Wolfgang Wagermaier, Khashayar Razghandi, Peter Fratzl

**Affiliations:** ^1^ Department of Biomaterials Max Planck Institute of Colloids and Interfaces Am Mühlenberg 1 14476 Potsdam Germany

**Keywords:** active materials, adaptive, bioinspiration, biological materials, responsive

## Abstract

The article explores materials sustainability through a bio‐inspired lens and discusses paradigms that can reshape the understanding of material synthesis, processing, and usage. It addresses various technological fields, from structural engineering to healthcare, and emphasizes natural material cycles as a blueprint for efficient recycling and reuse. The study shows that material functionality depends on both chemical composition and structural modifications, which emphasizes the role of material processing. The article identifies strategies such as mono‐materiality and multifunctionality, and explores how responsivity, adaptivity, modularity, and cellularity can simplify material assembly and disassembly. Bioinspired strategies for reusing materials, defect tolerance, maintenance, remodeling, and healing may extend product lifespans. The principles of circularity, longevity, and parsimony are reconsidered in the context of “active materiality”, a dynamic bio‐inspired paradigm. This concept expands the traditional focus of material science from structure‐function relationships to include the development of materials capable of responding or adapting to external stimuli. Concrete examples demonstrate how bio‐inspired strategies are being applied in engineering and technology to enhance the sustainability of materials. The article concludes by emphasizing interdisciplinary collaboration as a key factor for developing a sustainable and resilient materials economy in harmony with nature's material cycles.

## Graphical abstract

This perspective discusses natural materials as inspiration for sustainable engineering designs and the processing of materials. First, circularity, longevity, parsimony, and activity are presented as essential material paradigms. The perspective then uses many examples of natural and technical materials to introduce principles such as oligo‐materiality, multifunctionality, modularity, defect tolerance, remodeling, healing, adaptivity, as well as controlled disintegration.

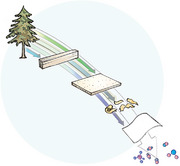

## 1 Introduction

Materials are at the core of technological development, providing the basis not only for buildings, machines, and functional devices but also for medication and health treatments. Materials are sometimes rare; they need to be extracted and purified or synthesized and processed. Materials are also often overabundant and constitute the bulk of waste that is either deposited or ends up in the oceans. Natural ecosystems use materials, from wood to skin and bone, but most of them are reused and/or recycled within ecosystems that can remain stable and self‐sustaining for a long time. Ecosystems generally comprise a wide range of organisms, including bacteria, fungi, plants, and animals that cooperate in the use, reuse, and recycling of matter.^[^ ^1^ ^]^ Human technology may be part of such cycles, for example when biodegradable materials are decomposed by microorganisms after human use.

The bulk of natural materials are based on a small selection of building blocks, mainly polysaccharides, proteins, and minerals. Different assemblages of these building blocks allow them to acquire a large variety of functions over many length scales, from the molecular to the macroscopic.^[^ ^2^ ^]^ One of the reasons for this limited selection is that biological systems operate at room temperature and environmental pressure conditions. Thus, they have no access to metal alloys or pure silicon, for example, because these require high temperatures and low vacuum conditions in their fabrication process.^[^ ^3^ ^]^ When exploring the areas of material composition and circular economy models, it becomes clear that a comprehensive understanding of the sustainable resource management achieved by natural structures can pave the way for innovative approaches to material use.

**Figure 1:**
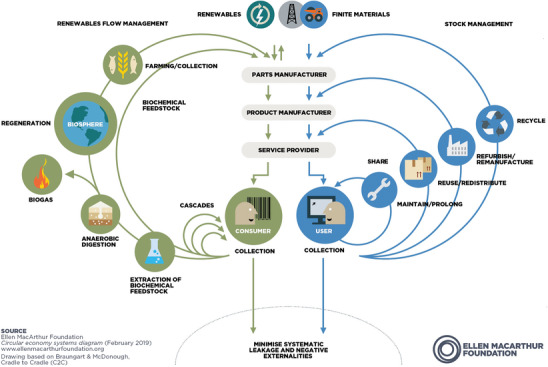
The butterfly diagram, a representation of the circular economy system, elucidates the perpetual material flow within a sustainable framework. The diagram outlines two principal cycles: the biological cycle, where nutrients derived from biodegradable materials are reintegrated into the Earth's ecosystem to support nature's regeneration; and the technical cycle, in which products and materials are maintained in circulation through practices such as reuse, repair, remanufacture, and recycling.^[^ ^4^ ^]^ Reproduced from Ellen MacArthur Foundation, 2024, www.ellenmacarthurfoundation.org, with permission.

The significance of a circular economy in bio‐inspired concepts is widely recognized today and often visualized in a butterfly diagram,^[^ ^4^ ^]^ as shown in **Figure** **1**. This comprises two fundamental cycles: the technical cycle and the biological cycle. Within the technical cycle, products and materials maintain circulation through practices such as reuse, repair, remanufacture, and recycling. Conversely, the biological cycle facilitates the return of nutrients from biodegradable materials to the earth, leveraging nature's regenerative processes. This diagram visually encapsulates the holistic approach of the circular economy, which promotes resource efficiency and environmental protection. However, the two cycles intersect for only a few activities, mainly in the areas of manufacturing and service.

**Figure 2:**
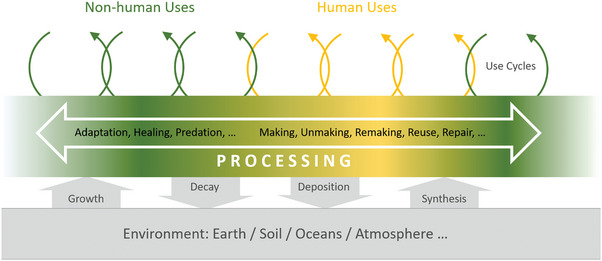
The key role of material processing for a more sustainable materials economy. Beyond chemical composition, tunable structures across length scales are key determinants of material functionality. Both natural materials and engineered processes create multiscale structures, linking the non‐human, natural world with human technologies. Precise material processing is essential for transferring nature‐inspired concepts to engineering, enabling a horizontal transfer of functionality based on shared building blocks. Reproduced with permission.^[^ ^5^ ^]^ Copyright 2023, American Chemical Society.

It may be appropriate to envisage a much broader interaction between these cycles (**Figure** **2**). A tree manufactures wood and leaves for its survival, while fungi and soil bacteria recycle dead plant material into nutrients for themselves and other organisms. Harvesting forestry products removes wood from this cycle, while biodegradation returns bio‐based materials to natural cycles. Agriculture and forestry have long considered these interactions, but fully separating other material cycles from natural ones risks significant environmental harm. Understanding the interplay between human‐created and natural material cycles is essential.

Therefore, quite apart from considerations relating to CO_2_ emissions and the associated climate change, making anthropogenic material cycles more sustainable will avoid wasting valuable resources and, at the same time, reduce the amount of waste that pollutes the environment. If natural material cycles can be an inspiration, then it becomes immediately clear that the problem cannot be solved from the viewpoint of materials science alone. It will be necessary to consider collaboration with other organisms, including plants, animals, bacteria, and fungi, as well as including all actors within the anthropogenic material cycles, such as production and processing, device fabrication, and recycling, as well as design, marketing, and usage (Figure 2). Thus, one of the prime goals of a sustainable materials economy will be to find better ways for all these actors to collaborate.

There is also a difficulty distinguishing unequivocally between materials and material systems. Indeed, defining the term “material” can give rise to complexities as it lacks a singular definition across all length scales. For example, substances like aluminum, polyester, clay, and wood can all be categorically termed as “materials” in the building block sense. However, in each instance, the defining characteristics of these “materials” manifest at distinct length scales. For instance: Aluminum is aluminum due to the comprising atoms, polyethylene is recognized by its polyethylene macromolecules, clay is defined by its ceramic hybrid building blocks and bonds, while wood is defined by its composite macro structure or more generally as a class of biomaterials. Lakes^[^ ^6^ ^]^ defined materials according to hierarchical levels, where the hierarchical order of a structure or a material may be defined as the number (n) of levels of scale with recognized structure. For n = 0, the material is seen as a continuum to enable the analysis of its physical properties; *n* = 1 (first‐order) could represent a latticework of continuous struts or the atomic lattice of a crystal.^[^ ^6^ ^]^ Going beyond the simple elemental view of the “material”, the term can be defined at any of these structural levels and depending on the problem or question at hand. This once again invites us to see material sustainability as a systemic problem that can be looked at and addressed at various scales.

Design processes primarily consider the potential functionality of materials within different systems, parts, or objects. The structure (organization) of these systems plays a crucial role in defining their relationships and functions within their environment.^[^ ^7^ ^]^ This relationship between organization and information at various length scales connects materials to the functions they serve in their assemblies. Consequently, the structure of materials serves as a bridge, linking the smaller scale compositions and elements to the larger context of functions and materials ecologies.

Our knowledge of the role of material architecture in biological material systems is increasing rapidly. In addition, new developments are being made in various fields of materials science and in the engineering and designing of matter. All this has provided us with contextual examples of how concepts such as responsivity, adaptivity, interactivity, self‐regulation, self‐healing, self‐degradation, multifunctionality, and mono‐materiality, to name just a few, can be understood and “programmed” through internal structuring, from the atomic level up to the meso‐ and macroscopic scales and in interaction with the environment.^[^ ^2^, ^7^, ^8^ ^]^

### 1.1 Current Approaches to Materials Sustainability

If materials are considered to be passive components of buildings and machines with a given set of properties, then their sustainable use is naturally described by three approaches: circularity, longevity, and parsimony.

#### 1.1.1 Circularity

Circularity encompasses strategies that facilitate the reuse or recycling of materials and structures. For example, many laws of physics are conservation laws that apply to mass and energy. Relating this to materials, we learn that – if we are aiming to reduce waste and save raw materials – we need to introduce reduction strategies and reduce the required material input, as well as introducing some kind of cycle in which materials are used and then collected for further use.^[^ ^9^ ^]^ As an example, metallic elements,^[^ ^10^ ^]^ rare earth elements, and petrochemistry‐based raw materials are becoming increasingly scarce, and their synthesis and production are energy and greenhouse‐gas intensive. Therefore, recycling as well as production methods with a low environmental impact can save primary resources and limit waste. Recycling breaks down materials to their base components for repurposing, while reuse preserves higher‐level structures, offering a more energy‐efficient path by avoiding full disassembly and reassembly. This reuse at higher structural levels supports circularity by decreasing the energy and resources needed to process materials from scratch, ultimately promoting a more sustainable and resource‐resilient future.

Circular sustainability strategies, which emphasize the importance of recycling and reusing materials and structures, have their roots in the universal qualities of materials. Parallel to this, the “universality‐diversity paradigm” illuminates fundamental structural elements and functional mechanisms present across a diverse spectrum of biological materials. Together, these themes not only reinforce each other but also provide a comprehensive framework for our discussion on sustainable material utilization, revealing how nature's universality in view of basic building units and materials enables circularity. The universality‐diversity paradigm was initially introduced for protein‐based biological materials,^[^ ^11^ ^]^ but it is essential for most natural materials that are not recycled within one line of application or even within a single species but rather by the complex interplay of many organisms in an ecosystem (see Figure 2).

#### 1.1.2 Longevity

Longevity covers a range of strategies that help materials and structures cope with changing or deteriorating conditions and prolong their functional life expectancy. It is closely linked to mechanical endurance, or more specifically to resistance, resilience, and robustness. When considering these three facets, we can draw inspiration from the properties we observe in biological materials, such as responsivity and adaptivity, cellularity and modularity as well as defect tolerance, remodeling, and healing. By linking examples from nature (section 2) to longevity policies, we aim to reveal how bio‐inspiration can contribute to material sustainability.

##### Resistance

Enhancing resistance in materials encompasses the concept of developing or reinforcing/adapting properties to effectively address changing or deteriorating conditions. This process often involves the evolution of responsive or adaptive systems to cope with environmental changes. Several examples illustrate this phenomenon: One such instance is observed in bone, where stress bridges are formed to precisely align with the lines of stress, effectively transmitting weight through the bone and increasing its strength. In trees, connecting roots serve as a strategy to withstand heavy wind loads, enhancing their stability. Furthermore, the formation of reaction wood in trees is triggered by external stimuli (e.g., strong continuous winds or loads through snow), resulting in a transition from regular wood formation to reaction wood formation across the hierarchical wood structure (see also Figure 7 in section 2.3 ). Transferring this concept to engineering or design applications, resistance can be increased by reinforcing structures. For example, welding parts of old bridges or infusing cracks in concrete columns can enhance their resistance and durability. Overall, the concept of resistance in materials encompasses multifaceted mechanisms that facilitate property development, adaptation, and reinforcement to ensure optimal performance and endurance in dynamic environments.

##### Resilience

Resilience in material systems can be defined as the ability to undergo temporary or pre‐yield deformations in order to withstand changing environmental conditions. This phenomenon can be categorized into two distinct types: (a) built‐in resilience within the material's structure and (b) responsive resilience through adaptive mechanisms. The built‐in resilience observed in certain materials can be exemplified by trees and grasses, which exhibit bending behavior in response to heavy wind loads. This inherent capacity allows them to absorb and dissipate energy from external forces, enhancing their ability to endure environmental stresses. Responsive resilience relies on dynamic adaptive responses to external stimuli. A noteworthy example of this can be found in mussel byssus threads, which possess sacrificial metal‐complex bonds (see section 2.6). Under mechanical stress, these bonds can reconfigure or break, absorbing energy and providing the thread with enhanced toughness and durability. This responsive resilience mechanism ensures that the mussel's byssus thread can adapt and protect itself in various marine environments. Understanding resilience in materials is crucial for the engineering of durable structures by harnessing built‐in and responsive resilience principles.

##### Robustness

Robustness in material systems can be defined as the capacity to withstand failure due to inherent material structures. A broader perspective of robustness incorporates the concept of “distributed functionality”, encompassing both cellularity and modularity (see section 2.4). One prototypical example is found in biominerals that exhibit a generic nanometer‐sized mechanical structure. This particle size, e.g., in mineralized tissues such as bone (see section 2.1), ensures optimal strength and maximal tolerance of flaws, rendering biomineral structures highly robust.^[^ ^12^ ^]^ Several other examples of robustness inmaterial systems can be found across various domains, e.g., due to cellularity in plant actuation systems. For instance, in the case of the ice plant's honeycomb keel, the system continues to function at (reduced) capacity even when several cells undergo degradation (see section 2.3 ). This inherent cellularity enables the plant to sustain its actuation processes despite the partial loss of cells, reflecting the robustness of the material system. A classic illustration of robustness due to modular, self‐similar structures can be observed in Roman streets that are made of cobblestones known as Sampietrini, which have been functional since the sixteenth century. The modular and self‐similar structure of these streets contributes to their exceptional durability and capacity to withstand loads and environmental impacts over centuries. In everyday design, the robustness of materials may be exemplified in a comparison of zippers and buttons serving the same general functionality. The modular design of zippers often necessitates replacing the entire zipper or major components if damage occurs, whereas buttons offer a greater degree of robustness through their simplicity, allowing for the easy replacement of a single button if it becomes damaged, which is generally simpler. Exploring the principles of robustness in materials and understanding in particular their structural features, cellularity, modularity, and distributed functionality can lead to more reliable materials capable of withstanding real‐world challenges.

#### 1.1.3 Parsimony

Parsimony comprises strategies that help to reduce the input of new materials into human use cycles. In our exploration of material sustainability, we will also take inspiration from biology by introducing the “good‐enough paradigm”, which encourages the prioritization of sufficient performance over the pursuit of perfection. Parsimony aligns closely with this concept since, in general, it emphasizes the minimization of material usage. Collectively, the concepts of parsimony and “good enough” offer a scholarly perspective on sustainable material practices, aligning with efficient and pragmatic strategies found in nature, as presented in section 2. Engineered Living Materials (ELMs) offer examples of materials that self‐repair and self‐sustain, aligning with principles of sustainability. By adopting design strategies that prioritize recyclability, decomposition, and resource efficiency inspired by nature's “good enough” principle, ELMs pave the way for more sustainable resource management in materials science.^[^ ^13^ ^]^

The concept of “good enough” can be elucidated through three distinct categories:

##### Addressing Over‐Optimization

The pursuit of perfect optimization hinders the recyclability of materials and systems throughout their life cycle. For example, wind turbine blades and certain packaging materials such as plastic‐coated paper cartons for food and drink may pose challenges in recycling due to their over‐optimized design. Exploring “sub‐optimal” solutions during the design phase could potentially mitigate such challenges.

##### Combating Compositional Over‐Diversification

The excessive diversity of function‐specific materials in various industries leads to resource‐intensive environmental burdens and recycling complexities. For instance, the metal industry's numerous compositional grades could be replaced by tuning properties through structure, reducing the need for various metal purities and compositions. This approach fosters parsimony and circularity in material usage by applying the principles of mono‐materiality and multifunctionality (see sections 2.1 and 3.1).

##### Leveraging Structural Modules

This approach involves recognizing the value of “sub‐optimal” structures that can function as recyclable modules applicable in other life‐cycle contexts. This shifts the focus from recycling individual elements to reusing entire structures, promoting sustainable material reuse and resource conservation (see section 3.2).

### 1.2 Active Materiality

**Figure 3:**
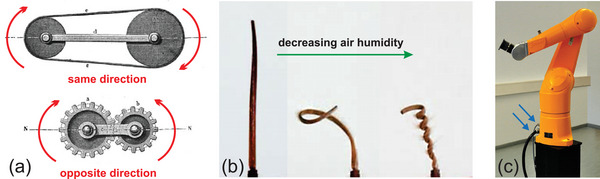
Degrees of freedom and energy supply in mechanical systems. a) The right wheel in the system governed by a belt or a gear has no degree of freedom. It has to turn either in the same or the opposite direction compared to the left wheel. The energy and information for the movement are transferred directly from the left wheel. Taken from: Kinematics of machinery: outline of a theory of machines (Franz Reuleaux, 1875), b) The various states of the Erodium awn depend on its water content, which in turn depends on air humidity. The information and energy for the movement are directly taken from the environment (changes in air humidity).^[^ ^23^ ^]^ c) In contrast to (a) and (b), the robotic arm in (c) has several degrees of freedom materialized by its articulations. The control is delegated to an outside processor and the information needs to be imported through a cable (blue arrow), as does the energy for the movement (second blue arrow). Reproduced with permission,^[^ ^16^ ^]^ under CC BY‐NC‐ND license.

In the design process, the focus lies on the potential functionality of materials, as function does not adhere to the same conservation law that governs passive materials.^[^ ^14^ ^]^ Material activity is defined as the capability to perform some work, either in a physical sense (measurable in energy per time) or in a symbolic sense, such as impressing mating partners or potential predators. Activity requires some level of information processing to transform a stimulus into a useful action. In some cases, this may imply conscious decisions, for example by humans, but in other cases, the information processing occurs directly in the material, based on a specific internal structure that codes for the useful response, such as in plant dispersal systems^[^ ^15^ ^]^ for example (see also **Figure** **3**). Thinking about materials as operators^[^ ^16^ ^]^ that transform an input (such as a mechanical load) to an output (such as a deformation) according to a material property (for example stiffness or strength), it becomes immediately obvious that chemical identity is not the essential descriptor of a material. A striking example are fibers, textiles or packaging foils, typically sold by length or surface area (in meters or square meters), emphasizing their essential function rather than their weight.^[^ ^10^ ^]^ The consideration of a specific context (system of interest) naturally directs attention toward the boundary conditions and constraints inherent to a particular system size. The concept of a material property depending on its constituents is challenged further by the development of metamaterials^[^ ^17^ ^]^ and, more generally, by architectured materials^[^ ^8^, ^18^ ^]^ that acquire sometimes unexpected properties based on the geometric arrangement of matter. This immediately relates to biological materials, which are generally architectured. However, biological materials also grow, adapt, and self‐heal,^[^ ^8a^ ^]^ which are all concepts transcending those of parsimony, longevity, and circularity. Since structure is an essential determinant of the property of materials and material systems, processing – understood as a procedure by which the structure of a material or a material system is modified – turns out to be an essential tool in connecting material cycles and improving sustainability (Figure 2). Indeed, in a recent collection of review articles on the chemistry of sustainable materials, processing appears as a common denominator.^[^ ^5^ ^]^

For the last few centuries, and throughout the period in which industrialization took place, materials have been viewed as passive elements to be formed and shaped into functional parts, objects, and larger systems. This industrialized “passivation of matter”, with its emphasis on building blocks, parts, and objects, is rooted in a need for the ever‐more reproducible, precise, and secure design of parts to serve specific functions for a certain lifespan. This hylomorphic view of the material world has continued into the electronic and the more recent digital industries of the present day.^[^ ^19^ ^]^

The current discourses around notions such as *active matter, active materials, animate materials, programmable materials*, and *life‐inspired materials* reflect upon a growing body of research from physics, biology, material science, and other fields that deals with a more active view of materials, adding a more complex, active, and interrelated layer to the passive “building block” view of materials.^[^ ^7^, ^20^ ^]^

Parallel to these developments in the natural sciences, there has also been a new revival in and revision of the materialism discourse from the humanities. This new materialism is a transdisciplinary effort stemming from various fields of anthropology, system theory, ecological sciences, information philosophy, cultural history, and further disciplines. It questions the established paradigm of materials as passive building blocks assembled from parts, objects, and other components. The emphasis in this new discourse is on a more active and entangled understanding of the material world.^[^ ^21^ ^]^

The first paradigmatic shift is that the new active matter discourse goes beyond the hylomorphic view, which had an elemental, compositional understanding of matter as a passive element ready to be shaped and formed. The view of matter as active recognizes the significance of material composition and places emphasis on structural aspects as being a crucial level of material description.^[^ ^7a^ ^]^

As will be discussed in the next section, the internal structure of plant seed awns allows for a degree of change based on the relative humidity of the environment. This illustrates the fact that information can be encoded directly into the structure and/or shape of a material. The seemingly old‐fashioned wheels in Figure 3 do exactly this. Their geometric relationship entirely determines the information that the wheel on the right receives about the direction in which it should turn when the wheel on the left turns clockwise. A seemingly more advanced process is illustrated by the robotic arm on the left, where all the energy for and information on its movement are imported via two cables. The plant seed awn (Figure 3a,b) takes both energy and information from its environment, essentially via temporal humidity gradients in the surrounding air. This underlines the importance of revisiting the paradigm of digitalizing industrial processes, which may consume more energy than needed in certain cases.^[^ ^16^ ^]^ Indeed, information processing in organisms does not always require centralization. The prime example are plants, which react and adapt to the environment without a brain, while information is processed in a delocalized way throughout the body.^[^ ^22^ ^]^ As shown by the example in Figure 3b, information processing in the material might in some cases use small energy sources from the environment and, thus, be more energy efficient than centralized external processing.

### 1.3 Objectives of this Article

This article introduces a novel approach to materials sustainability by exploring diverse biological materials, focusing on their unique properties and structural characteristics. It endeavors to conceptualize bio‐inspired augmentations of the three mentioned approaches—circularity, longevity, and parsimony—while also incorporating the concept of active materiality.^[^ ^14^ ^]^ This may enable a reconsideration of the discourses around material sustainability (see **Table** **1**).

**Table 1:** Outline of what we can learn from biological material solutions. The table displays the correspondence between a handful of categorized biological material implementations (vertical axis) and the proposed bio‐inspired material design paradigms (horizontal axis). The vertical axis is the “real‐world realization” of the horizontal axis, i.e., the implementation of these paradigms and principles in the form of biological materials. The paradigms can be realized in different ways and these options for realization in biological materials are indicated by the yellow boxes. Numbers in the table refer to sections where examples can be found.

Properties of biological materials	Circularity	Longevity	Parsimony	Activity
Mono‐materiality/oligo‐materiality	2.1, 3.1		1.1, 3.1	
Multifunctionality			2.2	2.2
Responsivity/adaptivity		2.3		2.3
Cellularity/modularity	2.4, 3.2	2.4, 3.2	2.4	
Controlled disintegration/reuse	2.5, 3.2		2.5	2.5
Defect tolerance/remodeling/healing		2.6, 3.3	2.6	2.6, 3.3

Section 2 will go through various biological material systems and present examples of the biological material implementations categorized in the vertical axis of Table 1. Section 3 presents some bio‐inspired material examples and concepts that have found their way, to varying extents, into materials engineering. Overall, we present concepts and potential tools for bio‐inspired approaches that would allow a reduction of raw material usage and waste, promoting a self‐sustaining, sustainable, and resilient materials economy.

## 2 Biological Material Implementations

This section reviews how different structural features, functions, and concepts of biological materials could potentially enhance the sustainability of technical material use. The examples chosen from nature illustrate design patterns and concepts that increase sustainability in the use of materials by organisms. The principles and implementations that will be discussed include: (i) mono‐ and oligo‐materiality to reduce the variety of materials, (ii) multifunctionality, responsivity, and adaptivity to tailor material structures in view of functions, (iii) modularity and cellularity to promote the simplification of material assembly and disassembly, (iv) the reuse of materials and structures to optimize materials and product cycles and, finally, (v) defect tolerance, maintenance, and remodeling as well as the healing and repair of materials to enhance the longevity of products.

### 2.1 Hierarchical Structures, Mono‐ and Oligo‐materiality

In biological materials, properties and functions are achieved through different types of structuring at various length scales,^[^ ^14^, ^24^ ^]^ rather than tuning and tailoring the chemical composition. Biology uses only limited resources. Through the evolutionary course of life, organisms have to make do with the affordances and resources available to them. From compositional and thermodynamic perspectives, this implies that biological material solutions must be derived from a relatively narrow range of available elements in the periodic table and be produced at low temperatures and pressures.^[^ ^3a^ ^]^

**Figure 4:**
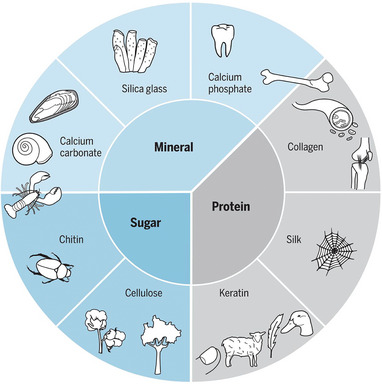
Biological materials are built with a limited number of building blocks based on polysaccharides, proteins, and minerals. A diversity of structures leads to a diversity of functions in tooth, bone, artery wall, tendon, spider web, beak, feather, wool, fingernail, tree, cotton, beetle carapace, lobster shell, snail shell, mussel shell, and the skeleton of the glass sponge (clockwise from top). Reproduced with permission from AAAS.^[^ ^2^ ^]^

The various biological materials are mainly the result of structuring three basic constituents: minerals, proteins, and sugars, in different ways, as shown in **Figure** **4**.^[^ ^2^ ^]^ Irrespective of the specific biological material, for example cuticle, bone, wood, or shell, a myriad of design features, such as layering, overlapping, fiber patterns, gradients, cellular arrangements, helical motifs, and tubular formations, facilitate the organization of these fundamental constituents across different length scales. This intricate structuring enables the realization of a diverse array of material architectures, properties, and functionalities.

Mono‐ or oligo‐materiality refers to the design or composition of a product or object using a single (or only a few) type(s) of material rather than a combination of different materials. The strategy of mono‐materiality in nature is often driven by the need for various functions of/in organisms (animals, insects, plants) and, at the same time, the limited availability of complex materials or combinations of materials to fulfill all these needs. Mono‐materiality is a strategy that facilitates the recycling of materials. Limited or reduced variability, together with the high purity of recycled materials, enhances the efficiency of recycling processes. This concept is already found in many industrial products and engineered materials: Paper (cellulose), steel, and many polymers are recycled if they have been appropriately separated. Mono‐material design of polymer materials uses chemically circular and biodegradable polymers derived from single monomers, enabling tailored properties through molecular engineering without altering chemical composition. Future advances depend on scalable production of bio‐based monomers, closed‐loop recycling processes, and adapting existing manufacturing infrastructure to process sustainable materials.^[^ ^25^ ^]^ Examples from nature often employs mono‐ or oligo‐materiality at different levels of hierarchy, and several aspects are still waiting to be transferred to technological processes and materials via bio‐inspired approaches (see section 3.1).

In this section (mono‐/oligo‐materiality) and the next (multifunctionality), we ask questions such as: Which types of materials can be considered to show mono‐/oligo‐material characteristics? Why and how are several properties combined in one material? Can material structures be employed to achieve various properties (e.g., mechanical or optical), or can mono‐materials even be made active and adaptive? We will discuss examples from nature (wood, bone, silk, and chitin) that are not made from single elements but still have mono‐material characteristics at a homogenous material level (see definitions of hierarchy from Lakes in section 1).

**Figure 5:**
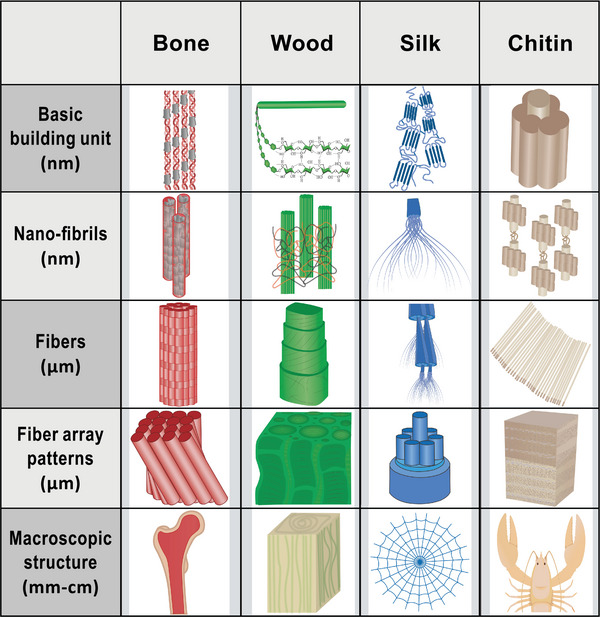
A schematic diagram of the hierarchical structure of: bone, wood, silk, and chitin. All materials exhibit a basic building unit at the nanometer scale and form fibrils and fibers in higher order structures (µm). Figure by Arun Kumar.

The hierarchical structure of natural materials, such as bone (collagen and mineral), wood (lignocellulose), silk (proteins), and exoskeletons (chitin and minerals), begins at the molecular level with a fundamental building unit that is prototypical for each material (**Figure** **5**). These materials share common structural elements, including fibrils at the nanometer level and fibers at the micrometer level, as well as diverse fiber array patterns at higher length scales. The specific arrangement of fibers contributes to the mechanical properties and strength of the materials, enabling them to fulfill their respective functions in nature. In particular, material properties are adapted to local needs by variations in one or several of the hierarchical levels without a modification of the chemical composition. A typical example is the variation of local mechanical properties by the variation of fiber orientations in bone or wood.^[^ ^15^, ^26^ ^]^

The basic building block of mineralized tissues (e.g., bone) is the mineralized collagen fibril, made from collagen molecules and nanometer‐sized calcium phosphate mineral particles.^[^ ^27^ ^]^ Although being a nanocomposite (hybrid material consisting of a soft collagen framework with hard mineral particles), bone represents mono‐material characteristics. At the micrometer scale, bone exhibits a rather homogeneous structure across larger areas when characterized by methods with a resolution limited to this scale (e.g., quantitative backscattered electron imaging). The intricate details of the nanoscale structure are not visible on the larger micrometer scale. As pointed out in section 1, it is difficult to define one single “material level”. Depending on the characterization method, we depict the mono‐material level in such a way that it is seen as a continuum to enable the analysis of representative physical properties. At a scale of a few micrometers, mineralized fibrils in cortical bone self‐assemble into fibril arrays forming the most common motif: lamellar bone.^[^ ^28^ ^]^ Again, variations in mechanical properties are closely related to the degree of orientation of the fibrils, in this case collagen fibrils. The hierarchical structure of the material of bone has also been shown to involve the presence of two differently structured material types in lamellar bone:^[^ ^29^ ^]^ the well‐known ordered arrays of mineralized collagen fibrils (Figure 5) and a second, relatively disordered material composed of individual collagen fibrils with no preferred orientation. The amount of ordered arrays far exceeds the amount of disordered material. In bone, these ordered and disordered materials have different local mechanical properties, which need to be understood in terms of the overall mechanical properties of the bone material in order to make it suitable as a source for bio‐inspiration.

In woody plants, the material is made up of (dead) cellulose tissue, which forms the secondary cell walls surrounding the wood cells. The cell walls are fiber composites made of cellulose microfibrils embedded in a matrix of hemicelluloses and lignin^[^ ^28^ ^]^ and at this level are viewed as mono‐material (Figure 5). These hollow tubes give mechanical stability to the plant, and the microfibril angle (MFA) of the fibrils in the secondary cell wall determines the mechanical and swelling properties of the macroscopic tissue, adjusting locally according to the biological or mechanical requirements. Depending on the age of the tree (young versus old) or the site (e.g., top versus bottom of a branch), the wood structure adapts its cellulose MFA to meet the required mechanical properties. The stem of a young tree (e.g., oak, spruce, or pine) is structurally adapted for flexibility while older trees become more and more adjusted for bending stiffness. These changes in properties are correlated to a decrease of the MFA from large values in the pith to smaller values toward the bark.^[^ ^30^ ^]^ There is a large microscopic diversity of wood structures with differently sized cells and pores, which is also essential for designing wood‐based materials in various contexts.^[^ ^31^ ^]^

Spider silk is a fibrous biological mono‐material (µm level) consisting almost entirely of large proteins.^[^ ^32^ ^]^ Spider silk fibers exhibit very good mechanical properties, such as strength and elasticity, resulting in outstanding toughness that is two to three times that of synthetic fibers such as nylon or Kevlar. The structure of dragline silk fibers reveals several hierarchical levels (Figure 5).^[^ ^33^ ^]^ On the molecular level, the primary structure of spider silk proteins is the amino acid sequence, whereas secondary structures form through intramolecular interactions and result in either alpha‐helical or beta‐sheet structures. Small crystallites (a few nm) are interconnected via an amorphous matrix with larger crystalline regions (>100 nm). In this two‐phase material, the amorphous matrix is responsible for the elasticity while the crystalline regions mediate the strength of the fiber. On the mesoscopic level, fibrils oriented along the fiber axis form the core of the fiber, resembling the structure of a rope. They are responsible for a more homogenous load distribution, ensuring optimal strength. On the micrometer level, the fiber shows a core‐shell structure.

Based on simple building blocks made from chitin and proteins, the exoskeleton of arthropods represents an example of a multifunctional material^[^ ^34^ ^]^ made from the same constituents at the lowest level of structural hierarchy, exhibiting mono‐material characteristics. The dominant material of the exoskeleton is the cuticle, made from chitin‐protein nanofibrils, which are protein‐decorated crystallites with a thickness of around 3 nm. Chitin units are comparatively stiff compared to the layers of soft and deformable proteins.^[^ ^35^ ^]^ Instead of varying compositions to achieve various functions, multiscale structuring and structural grading are two strategies leading to different properties within the cuticle. The mechanical properties of the cuticle are defined to a large extent by the chitin fiber architecture^[^ ^36^ ^]^ (Figure 5), which is mainly based on either unidirectional or helicoidal/rotated plywood structural motifs.^[^ ^37^ ^]^ In the exoskeletons of many arthropods, the fibrils are organized based on combinations of these motifs, resulting in different architectures that react differently to tension, compression, and shear stresses. The cuticle as a mono‐(hybrid) material therefore represents a prototypical example of how the organization of fibers can lead to a large variation in mechanical properties.

Biominerals are hybrid materials with an organic matrix enclosing an inorganic phase, typically calcium carbonate, calcium phosphate, calcium sulfate, silica, or silicate. As a common motif, they share a nanogranular structure, which is the main determinant of various properties at the mono‐material micrometer level of the biogenic ceramic material.^[^ ^38^ ^]^ The formation of building blocks in biominerals (e.g., nacre tablets, lamellae) is based on even smaller building blocks (e.g., mineral grains); thus, they build a composite material from a composite material or hybrid material of smaller scale. There are several mechanisms involved in the formation of biominerals: self‐organization, such as supramolecular template synthesis; template‐directed crystal growth; phase separation; and self‐assembly. These mechanisms have great potential for bio‐inspired materials chemistry and the formation of synthetic hybrid materials by tailoring structure, size, and properties from the nanometer to the macrometer level.^[^ ^39^ ^]^ Although this modular building block architecture is not designed for disassembly in biological materials (see section 2.4), it could improve recyclability if applied to complex synthetic materials.

### 2.2 Multifunctionality

Multifunctionality at the material level can be a strategy to reduce material variations and to enhance the longevity of products since it is often linked to rather simple mechanisms to achieve the required functions. Biological material systems frequently achieve multifunctionality through the intricate design of material structures, simultaneously achieving, for example, mechanical, optical, sensory, and other functionalities. This design is not necessarily limited to mono‐materiality as presented in the previous section. However, most biological material systems are restricted to a few basic elements or building units, with multifunctionality being achieved through smart material architecture. In this section, examples of biological materials will demonstrate the large variety of functions beyond mechanical properties.

Mineralized collagen‐based tissues, and in particular bone, represent a prototype for multifunctional biological materials. Bone is a living and dynamic tissue that maintains its functionality by modeling and remodeling. It is responsible for several vital functions in the living organism, such as providing support for soft tissues and enabling locomotion. In addition, bones enable the storage of minerals and growth factors and provide an environment for bone marrow.^[^ ^40^ ^]^

Bone incorporates pores of various sizes and shapes that are crucial for complex physiological functions. On the macroscale, long bones are hollow tubes, representing a confined space for bone marrow, which is the primary site of new blood cell production in mammals.^[^ ^41^ ^]^ The marrow's unique mechanical environment, influenced by external factors such as physical activity levels, governs pressure, shear stress, and viscosity, thereby affecting blood cell formation.^[^ ^42^ ^]^ Thus, the bone's essential role in blood cell production is vital for mammalian life.

At the micrometer scale and below, bone contains a further scale of porosity linked to vital functions: the lacunocanalicular network, which is a porous network of micrometer‐sized lacunae connected by canals with a width of several hundred nanometers known as the canaliculi. Osteocytes in lacunae can sense shear forces through their cell processes, which are located in the fluid‐filled pericellular space. As bone matrix deformations may be too small to be directly detected, a strain amplification mechanism using interstitial fluid flow is needed. This fluid flow induced by bone deformation triggers a mechanoresponse in osteocytes as they experience detectable shear forces.^[^ ^43^ ^]^ This complex function of sensing biomechanical forces and triggering bone remodeling is only realizable through the relationship between cell mechanosensitivity and the network architecture in bone. Another central function of bone is its role as a mineral reservoir during mineral homeostasis, regulated by the rates of calcium and phosphate entry and loss. Osteoblasts deposit and osteoclasts resorb mineral ions.^[^ ^44^ ^]^ Studies suggest that the osteocyte network also plays an active role in mineral homeostasis due to its dense architecture and therefore efficient access to large mineral reservoirs.^[^ ^45^ ^]^ Although these vital functions of bone can only be realized in the framework of living organisms, the role of the architecture of the bone material as well as of the porosities across several length scales is obvious. In view of transferring architectural principles to synthetically produced sustainable material systems, features like sensing systems via canalicular networks could play a crucial role, e.g., in self‐healing materials (see section 3.6).

Meta‐functionalities beyond the mechanical properties of wood (see previous section) include, for example, actuation as well as water and nutrition transport (see section 2.3). Its hierarchical structure and hydrophilic components facilitate efficient water and nutrient transport, which is crucial for plant survival. The vessels and tracheids within wood form open channels, enabling capillary‐driven water movement. Wood's fluidic properties extend to ionic transport, aided by aligned cellulose nanofibrils and hydroxyl groups that allow the tuning of surface charge properties.^[^ ^46^ ^]^ Optically, wood's brownish color comes from light absorption by lignin and light scattering by cell lumina, varying among wood species. Pure cellulose and hemicelluloses offer optical transparency, influencing wood's aesthetic appearance, especially in construction and furniture. Manipulating wood's composition and microstructure allows control over its optical properties.^[^ ^46^ ^]^ Wood's thermal properties, including its low conductivity and anisotropic heat transport, result from structural elements such as aligned cellulose nanofibrils and oriented lumina. Adjusting these thermal characteristics is possible by modifying the lignocellulosic composition and structural organization.^[^ ^47^ ^]^ Moisture content significantly influences wood's intrinsic thermal properties, impacting heat capacity and conductivity.

Spiders utilize the unique material properties of silk to construct geometrically organized web structures that serve many different functions, most significantly the effective capture of prey as well as the construction of egg sacs and cocoons. Beyond its superior mechanical properties (see section 2.1), spider silk shows further properties that might be a source for bio‐inspiration. It is adhesive, antimicrobial, biodegradable and biocompatible, sustains temperatures up to 200 degrees, and is able to realize “super contraction”, a shrinkage phenomenon if exposed to water resulting in a shrinkage in length of around 50 percent.^[^ ^48^ ^]^ The production of silk‐like materials through the molecular engineering of recombinant DNA into new primary structures allows the production of materials with high toughness or biocompatible materials for biomedical applications, for example.^[^ ^49^ ^]^ This bio‐inspired molecular engineering approach is well suited to forming actual building blocks for new sustainable materials with tailored properties (see section 3.2).

**Figure 6:**
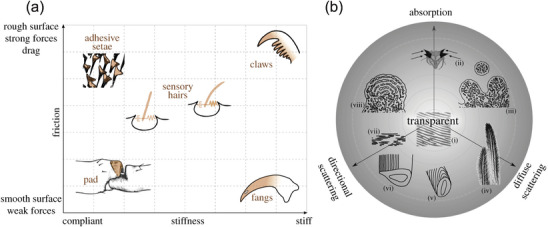
Illustration of the multifunctionality of spider cuticle and potential design spaces: a) Mechanical design space, contrasting friction and stiffness. b) Optical design space, represented as a cone projection with various structural examples: i) Transparent properties in spider cornea. ii) Melanin‐containing cuticular extensions in P. rubroargentea. iii) Yellow hairs of P. metallica with short‐range order domains. iv) Morphology of yellow hairs in P. metallica. v) Diffraction grating on M. nigromaculatus scale. vi) Diffraction grating on M. robinsoni scale. vii) Guanine platelet crystals in spider eye tapetum. viii) Blue hairs of P. metallica with ordered cuticular/air lamina. Reproduced from^[^ ^34^ ^]^ published by the Royal Society under CC BY 4.0.

The multifunctional cuticle of arthropods not only offers skin‐like protection but also enables locomotion and acts as a support structure for several tools for piercing, cutting, and interlocking, for the reception, transmission, and filtering of sensory information, as well as for structural colors, transparent lenses, and light manipulation.^[^ ^34^ ^]^ Therefore, it exhibits numerous functions in addition to its mechanical characteristics. Politi et al. employed a material science approach (Ashby map) to categorize variations in properties as a function of structural differences.^[^ ^34^ ^]^ This approach allows for the description of a mechanical design space, contrasting friction (surface property) versus stiffness (bulk property), as well as an optical design space. **Figure** **6** illustrates the material properties in different regions of the mechanical design space of the spider cuticle as a function of friction and stiffness.^[^ ^34^ ^]^ In the upper left corner, we find the sticky and compliant adhesive setae. In the opposite (lower right) corner, the spider fang represents an example of a stiff attachment employing a smooth surface to reduce friction. The spider claw (upper right) is rather stiff and used for attachment to the substrate by interlocking. Hair‐like structures are superhydrophobic and compliant. Auxiliary structures of the spider's highly sensitive mechanosensors include multiple substructures with varying mechanical properties that are made of the same basic cuticular material. These sensors promote biomechanical filtering, where information from stimuli is pre‐processed mechanically before being transmitted to the nervous system.^[^ ^50^ ^]^

Spiders use colors for intra‐species communication, thermoregulation, mimicry, and other functions and tasks. The cuticle's structural hierarchy creates optical effects and therefore opens up an optical design space. Figure 6b illustrates this with a 3D cone projection, where transparency is at its tip (i.e., in the center of the circle), and three axes extend to represent absorption, diffuse‐, and directional‐scattering.^[^ ^34^ ^]^ The arrangement of hairs and scales on a spider's body determines its overall spectral appearance. The color‐producing structures’ shapes govern iridescence at the micrometer scale. At a smaller scale, the organization and periodicity of substructures on setae and scales influence the purity (or bandwidth) of reflected light. In addition to spiders, the chitin‐based exoskeletons of other arthropods are also examples of multifunctional materials with specific mechanical and optical properties. The flower beetle, *Torynorrhina flammea*, shows how micropillar‐reinforced photonic multilayers simultaneously enhance mechanical robustness and optical damage tolerance, i.e., help preserve its visual appearance.^[^ ^51^ ^]^

Chitin‐based objects can not only be tuned to change their optical appearance but also to enable vision in animals, which is a dominant sense. Arthropods have chitin‐based compound eyes with a cuticular cornea that guides the light toward the light‐sensing unit. One prominent example of such compound eyes is found in *L. polyphemus*, the Atlantic horseshoe crab. Spaeker et al. found that refractive index gradients in the *L. polyphemus* cornea are influenced by structural and compositional factors such as chitin‐protein ratio, bromine doping, and water content variation.^[^ ^52^ ^]^ The structural variation is tied to the cornea's helicoidal chitin‐fiber architecture, which is derived from proteins around the chitin nanofibrils. This suggests that common mechanisms for adjusting mechanical properties also modify optical properties such as the refractive index.

The examples in this section represent, in one way or another, characteristics of multifunctionality, which allow—if transferred to synthetic materials—the reduction of material variations and increased product longevity by achieving functionality through particular structural arrangements and relatively simple mechanisms. The next section presents more complex mechanisms in biological materials, i.e., the way in which materials interact with external stimuli.

### 2.3 Active Materials: Responsivity and Adaptivity

Responsive and adaptive materials are characterized by dynamic material interactions with external stimuli. Responsivity and adaptivity correspond to different complexity levels in terms of material (re)action (Aizenberg in^[^ ^14^ ^]^ and^[^ ^53^ ^]^). A material is “responsive” when something changes in the material during exposure to a stimulus, be it temperature, humidity, light, pH, or a magnetic field. Adaptive materials add another level of complexity, i.e., the stimulus‐induced change in the material encounters a competing reaction and the output results from balancing the two via mutual feedback. Therefore, the response is adaptive, and the internal feedback enables complex material behavior.

**Figure 7:**
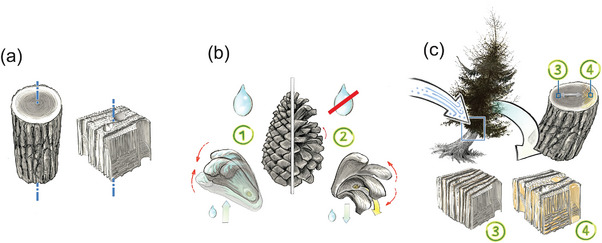
Principles and characteristics of responsive and adaptive materials. Any material with a certain property is an operator that transforms the input from the environment into an output. This is exemplified by the material wood. a) A tree stem exposed to a load deforms under compression. The elastic modulus is the material property that defines the deformation (output) as a function of the applied load (input). The elastic modulus is determined by the microstructures within the annual rings, such as the direction of cellulose microfibrils. b) In a pine cone, the direction of cellulose microfibrils is deposited in such a way that the cone is closed if hydrated (1) and opens on dehydration (2), so that the cellulose orientation can be considered a programming of the material for this specific purpose. c) Example of adaptation where the tilting of the tree after a landslide leads to different loading (compression) on the lower side than on the upper side where tension dominates. This results in depositing different types of reaction wood on both sides, leading to an asymmetric cross‐section of the stem. Different cellulose orientations on the upper and lower sides lead to different forces upon dehydration that generates a moment that will slowly reorient the stem into the vertical direction. This effectively constitutes a reprogramming of the wood material properties in response to the tilting event, which corresponds to an adaptive feed‐back loop. Reaction wood (with denser layers (3)) forms in the newly developed growth rings, leading to an asymmetric stem cross section that also has wider distanced growth rings (4). Figure by Konrad Eyferth.

Plants exhibit responsive and adaptive behavior and are good examples of how this can be embedded in the material itself. Responsivity can be conceptualized as an integrated “sense‐action” process within a material system.^[^ ^7a^ ^]^ When a property of a material is manipulated or generated through external stimuli, the dynamic between input and output is altered, enabling the material to be programmed. Such systems are commonly denoted as responsive materials. If the adjustment of the property is influenced by the output level, it gives rise to a feedback loop, and the material is thus adaptive. In **Figure** **7**, these material concepts are exemplified in wood in the following manner: (i) A stem or solid piece of wood exhibits a certain strength (property), and load (input) is transferred across the material as another load (output). (ii) Pine cone scales exhibit a reversible hydro‐responsive behavior, opening and closing in response to water. This is due to their programmed material structure triggering a shape change. The perpendicular orientation of cellulose microfibrils in the two different scale layers causes varying dimensional changes during water absorption, leading to outward and inward bending of the scales during drying and wetting cycles. (iii) Trees usually grow vertically, but here a softwood tree stem has been impacted by either snow or scree, resulting in a tilting of the rootstock and therefore the formation of reaction wood (different layer thickness) due to adaptive growth.

An additional straightforward illustration of material adaptivity is found in our muscular and skeletal systems. They can undergo augmentation in thickness and strength, resulting in heightened response to various stimuli. For instance, engaging in physical exercise imparts a mechanical load – embodied by the weight being lifted – as the input. This input correlates with the output, defined as the maximum weight that can be lifted. The interconnection between these parameters is mediated by the muscle's mechanical contraction force, contingent on its cross‐sectional area, which notably amplifies with the progression of training. In such adaptive systems, the output can be modified through positive or negative feedback loops.^[^ ^53^ ^]^ Homeostatic systems work with negative feedback loops in which an increase in the output leads to a property modification to reduce the output and stabilize the system. Homeostatic processes in living systems preserve the amount of material despite varying external challenges. However, in positive feedback loops, the effect of the output on the system property is such that it enhances the output, leading to the destabilization of the system. Moving away from a stable equilibrium state can lead to possible failure, but it can also be productive and push the system into a new homeostatic equilibrium, for instance to induce growth.

Plants are also able to utilize water for stress generation and for movements that are essential to several mechanisms, such as growth, spatial orientation, acquiring nutrition, or seed dispersal (Razghandi et al.^[^ ^14^ ^]^). Hydro‐actuated movements are based on complex interplays of the material structure at various molecular, cellular, tissue, and organ scales. The snapping closure of the leaves of the Venus flytrap is one of the fastest movements among plants (approx. 100 m s^−1^). Insects touching the sensitive hairs of the leaves cause a biochemical response, inducing a water flow and the inflation of cells. The resulting differential volume change of the leaf tissue leads to a rapid morphing from a convex to a concave shape and thus the closing of the trap. An innovative example of hydro‐actuated movement is a light‐driven artificial flytrap that autonomously closes and recognizes objects.^[^ ^54^ ^]^ Using a light‐responsive liquid‐crystal elastomer integrated onto the tip of an optical fiber, this device combines photomechanical actuation with environmental sensing, mimicking natural flytraps. This fiber‐sized design enables self‐regulated actuation, showcasing potential for soft, autonomous small‐scale devices. Similar to pine cones,^[^ ^55^ ^]^ wheat awns^[^ ^56^ ^]^ are also cellulose‐based mono‐materials that exhibit hydro‐stimulated actuation; a bending movement during drying and rewetting is achieved by a bilayered structure. In both systems, opposing tissues possess different cellulose fibril orientations in their cell walls. Ice plant seed capsules can achieve even more complex actuation due to a swellable cellulosic layer surrounded by a non‐swellable honeycomb framework.^[^ ^57^ ^]^

### 2.4 Cellularity and Modularity

The terms modularity and cellularity describe structures with repeating units, that is, the modules or cells. Modules are units in a larger system that are structurally somewhat independent of each other but work together.^[^ ^58^ ^]^ Therefore, modular systems provide a framework (architecture) that allows for both independence of structure and integration of function. Modular design may significantly enhance materials sustainability since lightweight modular structures contribute to the aspect of *parsimony*, and modularity facilitates the replacement of sub‐units, which consequently contributes to better *recyclability* as well as to the overall *longevity* of products due to repairability. In general, modularity aids bottom‐up construction and self‐organization across multiple length scales, ultimately also yielding emergent material and structural properties. Emergent properties are macroscopic characteristics derived from microscopic interactions and organization; they are not predictable solely from individual component properties.

Modularity in biological materials starts at the molecular length scale with amino acid monomers but is also found at higher order levels throughout hierarchical structures. A repetitive modular design exists in many structural proteins, including elastins, collagens, and fibronectins.^[^ ^59^ ^]^ At larger length scales, modular structures are for example found in honeycombs, wood cells, and tessellated cartilage in sharks and rays, as well as in many more organisms.

**Figure 8:**
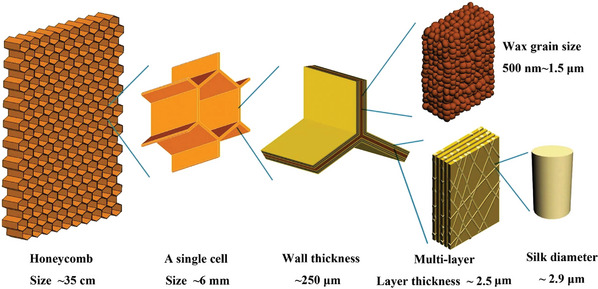
Hierarchical structure of one‐year‐old honeycomb at macro‐, micro‐, and nanoscales. At the macroscale, there are the honeycomb structure and the single cell; at the microscale the multilayered cell walls and the wax grains; and, finally, the silk fibrils at the nanoscale. Reproduced with permission.^[^ ^61^ ^]^ Copyright 2010, National Academy of Sciences.

Honeycomb structures are one prominent example of modular and cellular structures in nature. Bees build honeycomb in the form of hexagonal prismatic wax cells in their nests to store honey and larvae. The honeycomb structure is based on repeated units (closed cell structures) taking the form of uniformly distributed double‐layered hexagonal cells.^[^ ^60^ ^]^ Honeycomb exhibits a hierarchical structure (**Figure** **8**), and its walls are made from wax. Over time, they are continuously strengthened and stiffened by the addition of thin wax layers reinforced by silk fragments that are recycled from larvae cocoons.^[^ ^61^ ^]^ Natural honeybee combs are often presented as an archetype for the engineering of cellular structures, focusing only on their macroscopic geometry. However, honeycomb structures exhibit multifunctional characteristics, including lightweight construction, thermal insulation, and energy absorption. Zhang et al. state^[^ ^60^ ^]^ that applications of man‐made honeycomb focus primarily on: (i) ultra‐lightweight design to achieve simultaneously high strength and low density, e.g., in architectural, automobile, and aerospace structures, (ii) high impact energy absorption applications such as armor and other shields, and (iii) high macroscopic shear yield strength, e.g., in non‐pneumatic tires. However, at the nano‐ and micrometer scale, natural honeycomb exhibits a hybrid and composite character. In view of sustainability, it even involves aspects of recycling (silk from cocoons in the wax walls). Therefore, it is a rich source of inspiration for materials engineers and designers interested in sustainability.

Tessellated structures are materials with a repeating structural unit (modules). Tessellation describes a structural motif that involves periodic soft and hard elements arranged in series and that appears in many invertebrate and vertebrate animal biomaterials at several length scales.^[^ ^62^ ^]^ This combination of hard elements and relatively soft connecting layers provides a range of interesting mechanical properties, including the prevention of crack propagation, flexibility, and hardness for biological coatings and armors, and even strain enhancement and signal filtering for mechanosensing. There are many examples of tessellation in nature (see: https://tessellated‐materials.mpikg.mpg.de/collection), ranging from prismatic layers of mollusk shell,^[^ ^63^ ^]^ which comprise columns of mineralized rods surrounded by an organic protein layer, to tessellated cartilage in sharks and rays,^[^ ^64^ ^]^ which features a mineralized layer covering the skeletal cartilage, as well as other examples such as reptile scales^[^ ^65^ ^]^ comprised of keratin, which are connected by an elastic protein tissue. Another prominent example of tessellated structures are turtle shells, which serve as armor providing varying degrees of protection against predation.^[^ ^66^ ^]^ The shape of the suture between adjacent bone segments provides for easy deformation at small loads and results in a much stiffer shell due to the locking of the neighboring bone segments.^[^ ^66a^ ^]^

The building blocks found in hierarchically structured biological materials are another example of a modular concept; they have the characteristic of being repeating units within more complex material structures. Interfaces between building blocks enable certain functionalities and enhance certain properties, such as improving material toughness, joining materials of different character, allowing them to deform more easily, and even enabling actuation.^[^ ^67^ ^]^ The interfaces represent only a very small fraction of the overall volume but are essential for the integrity and function of the whole tissue. In the next section, the role of interfaces in biological materials will be discussed in terms of both their assembly and disassembly capabilities.

Engineered Living Materials present a promising bioinspired pathway for sustainable materials, offering alternatives to high‐performance materials whose complex processing often limits recyclability.^[^ ^13^ ^]^ Unlike traditional materials, which are challenging to decompose and recycle, ELMs are designed to self‐repair, self‐support, and grow, reflecting Nature's principle of using local resources and achieving functionality with “good enough” efficiency. While technical materials provide unparalleled durability and precision, ELMs offer adaptability and environmental compatibility that can reduce waste and extend material life. Integrating ELMs with engineered advantages, such as modular component reuse, could enhance sustainability by blending natural principles with human‐made precision, paving the way for material systems that support both performance and environmental health. Engineered living materials incorporating genetically engineered microorganisms for user control, can produce their own matrix (e.g., biofilms) or be integrated into matrices using technologies like 3D printing, coating, spinning, and microencapsulation.^[^ ^68^ ^]^ Such ELMs hold promise for biomedical applications, including biosensing, wound healing, tissue engineering, and drug delivery.

### 2.5 Self‐Assembly and Self‐Organization

The controlled disintegration of material structures or their simplified disassembly into recyclable (mono‐material) components can make a relevant contribution in making materials more environmentally friendly. Related concepts in biological materials include: 1) structure‐function relations facilitating ease of disassembly, 2) reversible bonds for disassembly (e.g., resorbable glues), and 3) mono‐ and oligo‐material structures contributing to the ease of disassembly and recycling, as observed in biological hybrid materials such as mineralized collagen fibrils.

Self‐assembly involves processes at a single level without adaptive capacities, leading to the formation of ordered patterns. Self‐assembly refers to the process by which numerous individual entities spontaneously associate to form organized and well‐defined structures, driven by inherent interactions and without requiring external guidance.^[^ ^69^ ^]^ In contrast, self‐organization is associated with dynamic processes and dissipative structures featuring multiscale interactions and synchronization. It enables adaptation, reparation, resilience, and the emergence of properties in the system. At the molecular level, the reversibility of noncovalent interactions between molecular building blocks such as amino and nucleic acids, proteins, and lipids determines the formation as well as disassembly mechanisms of biological materials. At higher‐order structural levels, building blocks (e.g., mineralized collagen fibrils in bone, cellulose fibrils in wood, and chitin fibrils in cuticles) are often held together by the same noncovalent interactions. The reversibility and adaptivity of noncovalent interactions enable new functions that are not available to their covalent counterparts, one of which being the controlled disassembly of building blocks in materials.

For example, reversible bonds in biological materials are found in bone and other mineralized tissues in the form of so‐called sacrificial bonds, which are noncovalent interactions and allow a certain degree of deformation without damage. Sacrificial bonds greatly increase the fracture toughness of biological materials by providing a reversible energy‐dissipation mechanism.^[^ ^70^ ^]^ Above a certain load, weak reversible bonds are the first to break, allowing covalent chains to unfold and consequently facilitate substantial deformation to the material without disrupting the chain.^[^ ^62^ ^]^ Although these types of bonds in biological materials are not usually intended to serve the purpose of dissembling the complex structured materials, their reversible nature can be employed to break materials into parts that are either mono‐material in nature or at least easier to feed into a recycling process.

The reuse of wood after its primary purpose has been completed or the recycling of wooden structural elements at higher hierarchical levels plays a pivotal role in minimizing waste and fostering sustainability. In nature itself, this concept is exemplified by incorporating wooden parts into other natural structures, as seen in birds’ nests and other ecological processes. Wood reuse in both human practices and natural systems contributes to more efficient resource utilization. Waste is a problem of linear industrial processes and barely occurs in circular biological processes. Natural processes do not usually produce waste that represents an environmental problem; rather, it is an intermediate step of circular processes, and discarded elements are reused to form new materials and structures. While waste is minimal in many circular biological processes, examples like biomineral deposits illustrate exceptions where natural processes generate waste material. For instance, diatom frustules, coral skeletons, and coccolithophore shells can accumulate as sedimentary deposits, significantly influencing ecosystems and global biogeochemical cycles (see also the relation between biological cycles and the environment in Figure 2).

Another example of disintegration and reuse is evident in soil ecosystems, where organisms participate in decomposing organic matter to varying extents, breaking down complex structures into simpler components. However, not all components undergo complete disintegration to the level of individual molecules or atoms. Plant litter and microbial biomass are key sources of soil organic matter formation.^[^ ^71^ ^]^ Plant litter is a complex mixture containing cellulose, lignin, and other biopolymers. It contains varying types and amounts of plant species and tissues. Soil microorganisms, including bacteria and fungi, initiate the decomposition process by enzymatically breaking down complex compounds such as plant residues and animal remains. Some organic compounds show resistance to microbial degradation due to their robust structures. These compounds persist in the soil and serve as valuable substrates for subsequent biological processes that are crucial for nutrient cycling, microbial interactions, and soil fertility. Consequently, they contribute to the formation of humus. Recent advances have demonstrated that artificial humic substances, synthesized from waste biomass through alkaline hydrothermal processing, can enhance soil structure, improve water and mineral binding, and support plant growth.^[^ ^72^ ^]^ While the direct impact of artificial humic substances on soil properties appears minimal, its influence in terms of stimulating beneficial soil microbiomes is significant. By integrating natural and synthetic humic substances into the concept of engineered living materials, this approach leverages traditional chemical principles such as surface activity, redox mediation, and pH buffering to create adaptive, hybrid systems. These hybrid systems offer targeted solutions for enhancing soil fertility, promoting sustainable agricultural practices, and mitigating climate change impacts through improved carbon sequestration and ecosystem resilience.

### 2.6 Preventing Failure: Defect Tolerance, Remodeling, and Healing

Damage and fatigue are omnipresent in natural as well man‐made materials. Cracks, fissures, and defects appear in materials even during normal use at some point during the lifespan of an object.^[^ ^73^ ^]^ However, living organisms routinely produce long‐lasting materials, which is often a result of their remarkable ability to either sustain or remove defects before material failure (defect tolerance and remodeling) or to recover material functionality after failure (repair and healing). Defect tolerance and remodeling are preventive measures in materials and represent design strategies to increase the longevity of materials through failure prevention. These strategies have in common that materials are damaged to some extent but are still able to fulfil their functionality. Defect tolerance means that materials accumulate defects but do not fail. Remodeling is a strategy to remove defects in materials, and the mechanism is based on the dissolution of components to remake them without defects.

Bone has repeatedly been presented as a blueprint for a material simultaneously exhibiting strength and elasticity that combine into relatively high toughness. The reason behind this is found in its complex hierarchical structure, as described in section 2.1, but also in its defect tolerance and a multitude of toughening mechanisms across several length scales,^[^ ^26^, ^74^ ^]^ as well as in continuous structural maintenance due to remodeling.

**Figure 9:**
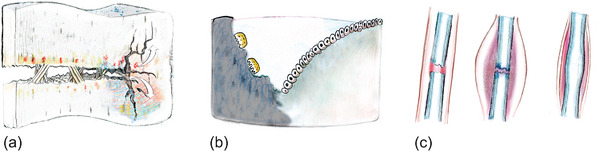
a) Defect tolerance, demonstrated on the example of toughening mechanisms in bone. A crack running through bone may be hindered by, e.g., crack bridges (fiber bundles) or crack deflection due to lamellar structures (cracks run in different directions). b) Remodeling in bone: bone‐resorbing cells (osteoclasts) remove old bone (left) and bone‐forming cells (osteoblasts) rebuild new bone tissue (right) without loss of overall bone functionality. c) Healing in bone, displayed in three different phases: a broken bone (left) starts the healing phase by callus formation (center), followed by remodeling (right), which all together restores structural integrity of the bone. Figure by Konrad Eyferth.

Defect tolerance is the capacity of a material to maintain strength even under the presence of cracks or flaws and is an essential requirement in the design of composite materials.^[^ ^75^ ^]^ **Figure** **9**a shows a simplified version of a crack running through bone material and two related mechanisms increasing bone toughness. The crack opening is hindered by crack bridges (fibers crossing the crack) as well as by the lamellar structure of bone at the micrometer level. This deflects the crack (see the scattered small cracks at the crack tip), and more energy would therefore be required to open further crack surfaces. These mechanisms allow for the absorption of energy (introduced by load) and thus prevent bone fractures. Since bone undergoes continuous remodeling, the microcracks that absorbed energy during their formation are removed during the remodeling cycles (Figure 9b). In bone and nacre, studies on deformation and fracture have shown the importance of molecular interactions and bond recovery (see also section 3.5).

Remodeling is a mechanism through which bone can adapt to a changing mechanical environment and involves the continuous replacement of old bone material with new.^[^ ^28^ ^]^ This process happens via a sequence of cellular events occurring on the same surface, without any change in overall bone shape.^[^ ^76^ ^]^ The renewal of bone occurs during the life of the organism through an interplay between different bone cells: osteoclasts resorb bone and osteoblasts initially deposit an unmineralized matrix, which is then mineralized (Figure 9b). Some of the osteoblasts differentiate into osteocytes, being buried in the mineralized matrix. Thus, osteoclasts disintegrate the mineralized collagen fibrils by using biochemical processes to retrieve calcium and phosphate ions; these are recycled and appear again in the newly formed bone. This is also an essential part of mineral metabolism since bone serves as a reservoir for calcium and phosphate. Bone remodeling is therefore a circular biological process without waste. However, a living organism constantly consumes energy.

In contrast to the abovementioned preventive strategies that enable material failure to be avoided, in the case of a material failure, in nature strategies of healing and repair are often used. This also allows the longevity of the material to be increased and consequently can even increase the lifespan of entire organisms that depend on the functionality of particular biological materials.

Healing processes in living organisms depend on cellular activity in animals (e.g., bone healing, antler growth, and limb regeneration) as well as plants (e.g., latex‐based healing, plant grafting, and wound closure). Self‐healing and self‐repair result in the restoration of the original function of the material after damage. The structure is not necessarily completely restored in comparison to before the damage. Bone healing (see Figure 9c) is a dynamic and highly regulated biological process characterized by the sequential phases of inflammation, soft callus formation, hard callus formation, and remodeling; this restores the mechanical integrity and structural properties of the injured bone tissue through the coordinated actions of osteoclasts, osteoblasts, and various growth factors.

Self‐repair mechanisms in organisms are generally subdivided into two phases: an initial self‐sealing phase and a subsequent self‐healing phase. Sealing and healing are both characterized by anatomical and biochemical modifications and changes in biomechanical properties.^[^ ^73^ ^]^ There are several successful examples of biological self‐healing mechanisms: i) the latex‐based healing of plants, ii) cell‐based healing in pipe vines, and iii) reversible sacrificial bonding in extracorporeal biopolymers such as the mussel byssus. Marine mussels anchor to wave‐battered surfaces in rocky seashore environments using protein‐based holdfast fibers known as mussel byssal threads. When critical stresses are exceeded, yield results in apparent damage in subsequent loading cycles.^[^ ^77^ ^]^ However, the initial mechanical properties are largely recovered when threads rest for several hours in water, indicating an intrinsic self‐healing of material damage.

Plants demonstrate regrowth phenomena in their leaves, needles, and fruits, which are typically replenished during annual cycles. The shedding of leaves and parts of fruits is followed by the action of microorganisms that facilitate the disintegration process. As a result, a significant portion of these fallen organic materials transforms into compost, serving as a nutrient source for the parent plants that initially bore these leaves and fruits (see also section 2.5). This regenerative process exemplifies a natural and efficient nutrient cycling mechanism within ecosystems, facilitating sustained growth and the vitality of plant communities.

In summary, the manifold examples from nature presented in this section represent an endless source of inspiration for the design and engineering aspects of materials science. In addition, their potential mechanisms and properties can be categorized to enable a more systematic use of these principles in design and engineering. To answer the question of how materials, objects, and products can be made more sustainable based on strategies found in nature and in biological materials, we need to derive bio‐inspired principles related to structure, properties, processes, and usage. The next section is an attempt to categorize such principles, as found in biological materials; we depict them as bio‐inspired material design paradigms.

## 3 Bio‐Inspired Examples and Concepts

This section presents examples and concepts from engineering and technology that utilize bio‐inspired strategies to enhance the sustainability of material life cycles. These examples provide a partial illustration of a set of concepts that have already been realized in the area of material design or in the production of materials, structures, and products.

### 3.1 Bio‐inspired Mono‐Material Examples and Concepts to Achieve Multifunctionality

Mono‐material concepts in the manufacture of engineering materials can be fostered through the following three key paradigms: structure‐function, universality‐diversity (circularity), and “good enough” (parsimony).

**Figure 10:**
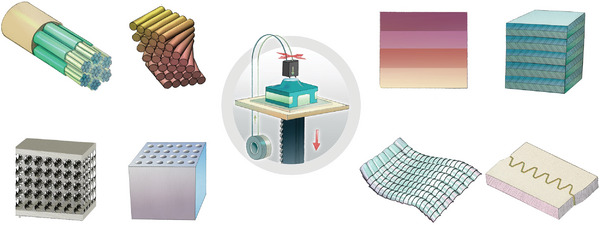
The eight most common biological structural design elements (from upper left to lower right): Fibrous: high tensile strength in one direction, minimal compressive strength; Helical: toughness in multiple directions, in‐plane isotropy; Gradient: gradual property transition to reduce interfacial stress, enhancing toughness; Layered: complex composites that improve toughness, especially in brittle materials; Cellular: lightweight porous structures for stress distribution and energy absorption, often in sandwich forms; Tubular: organized porosity for energy absorption and crack deflection; Overlapping: layered plates or scutes for flexible, often armored surfaces. In the center, a 3D printer is shown schematically; Suture: interdigitating interfaces for controlled strength and flexibility (figure inspired by: Naleway et al.^[^ ^80^ ^]^) Figure by Konrad Eyferth.

One prominent example of manufacturing objects using a mono‐material approach is layer‐by‐layer deposition of materials, better known as additive manufacturing (AM) or three‐dimensional (3D) printing.^[^ ^78^ ^]^ Single‐material AM is sometimes stigmatized as a limitation since it supposedly restricts the end‐use functionality of the fabricated structures due to their narrow material property ranges.^[^ ^79^ ^]^ However, in view of sustainable materials use, this feature can be turned into an advantage if bio‐inspired design is incorporated into the production process, for example with variations of structural patterns such as fibril orientation. Naleway et al. identified eight structural design elements from biological materials as common motifs (**Figure** **10**): fibrous, helical, gradient, layered, tubular, cellular, overlapping, and suture. These motifs may serve as a toolbox for rationalizing the complex mechanical behavior of biological materials and consequently promote the more systematic bio‐inspired design of artificial materials. Several of these structural design elements can be employed by means of AM to produce bio‐inspired geometrical and functional structures.

Lightweight cellular structures are rather simple examples of mono‐material constructions made by AM. In particular, the cellular structure of wood, with its variable mechanical properties, calls for a bio‐inspired reproduction of its structures and functions by means of AM techniques. There are several material options in AM for the formation of wood‐inspired cellular structures: (i) synthetic polymers, (ii) wood‐derived compounds such as nanocellulose, or (iii) other suitable mono‐materials, e.g., synthetic hybrid materials. 3D printing approaches allow the production of wood‐inspired composites with a tunable mechanical response, featuring a central layer with rigid helicoidal fibers embedded in a considerable softer matrix.^[^ ^81^ ^]^ A prototypical element could be structured and printed as follows: Fibers wind in a helical manner with respect to the principal longitudinal axis of the cylinder and, by modulating the orientation of the stiff elements in different layers as well as the ratio between the elastic modulus of fibers and matrix, the mechanical properties are adjustable in a way that is similar to natural wood. More specifically, the 3D printing of lightweight cellular composites with a controlled alignment of multiscale, high‐aspect‐ratio fiber reinforcement allows the creation of hierarchical structures inspired by balsa wood.^[^ ^82^ ^]^

Nowadays, many AM techniques go beyond the mono‐material approach. However, similar to nature, usually only a few different materials are combined in AM, turning it into an oligo‐material approach. Several bio‐inspired examples exist in this field, but a detailed review would go beyond the scope of this article. The combination of different materials in AM allows the creation of materials with locally tuned chemical compositions and bio‐inspired microstructures that are not accessible by conventional processing routes.^[^ ^83^ ^]^ This includes anisotropic building blocks with optimized size and aspect ratio, hierarchical structures over multiple length scales, the combination of strong and weak bonds between building blocks to enable dynamic functionalities (adaptation, remodeling, self‐healing), and the controlled spatial distribution of building blocks into heterogeneous or graded architectures.

AM brings about additional advantages in terms of sustainable material production. The free‐form fabrication nature of AM eliminates some of the design constraints of traditional manufacturing processes and enables an optimization of material structures by reducing the materials, energy, and other resources required in the product manufacturing process.^[^ ^84^ ^]^ Moreover, non­processed raw materials can be recycled and reused by AM to reduce material waste and therefore contribute to the circularity concept. AM can be performed at the customer's premises to maximize the efficiency of supply chains and consequently reduce the need for long‐distance transportation, warehousing, and disposable packaging. Finally, AM can be used to repair defective products to extend longevity.

The packaging of food is an example of multi‐material use within one product (multilayered packaging foil) due to multifaceted requirements, such as providing barrier properties against UV light and oxygen, which are the main causes of most food degradation processes.^[^ ^85^ ^]^ However, the combination of different materials in a single product through lamination or co‐extrusion turns the technical advantages obtained in terms of better performance into disadvantages in terms of recyclability. In nature, fruit peels provide an efficient, multifunctional barrier that protects food from mechanical damage and microbial threats, offering a valuable model for bioinspired packaging. For example, the pomelo peel's hierarchical structure—comprising a soft, elastic mesocarp for impact absorption and a dense exocarp for puncture resistance—has inspired sustainable composites for fruit preservation.^[^ ^86^ ^]^ Such biomimetic materials not only protect delicate foodstuffs from impact and puncture but also enhance preservation by incorporating natural antimicrobial agents, potentially extending shelf life significantly and reducing food waste.

However, the contribution of mono‐material concepts to circularity becomes even more significant if viewed from the perspective of the variety of different materials being used for similar purposes. To stay with the example of food packaging, the huge diversity of packaging materials used for different foods can be observed by visiting any supermarket. Most of the packaging is polymer based, but this often includes hundreds of combinations of various polymers. A significant contribution to easing the recycling of polymers would be to define the most important parameters for food packaging (e.g., mechanical properties, barrier properties, sustainability value) and derive only a few polymer materials that cover all these properties. This could be represented as a food packaging design space where, similar to Ashby maps, the parameters could be plotted against each other. Such a food packaging materials map would then allow the selection of the most appropriate polymer material from the perspective of both its material properties and in relation to sustainability.

Polymeric materials are essential for AM techniques, but their fossil fuel basis and poor degradability harm ecosystems. Developing sustainable biopolymers inspired by natural materials is crucial. Advances in gene sequencing, biotechnology, and synthetic biology have enabled the rapid design and production of protein‐based materials with precision. A recent review points out that future research will focus on enhancing microbial strains and bioprocessing methods to create effective, sustainable biopolymers, integrating high‐throughput screening, automation, and machine learning in order to optimize production and material properties.^[^ ^87^ ^]^ By focusing on biological models like spider silk, mussel byssus, and hagfish slime, nature's blueprints for creating greener fibrous polymeric materials can be utilized.^[^ ^88^ ^]^ It will be of paramount importance to identify common self‐assembly principles across these systems and to emphasize their potential for bio‐inspired material fabrication in areas such as liquid‐crystal phase separation and microfluidics. There are still challenges in mimicking biological processes, such as achieving spatiotemporal control and scaling up production. A comparative analysis of various biofibers has revealed the key design principles for sustainably assembling high‐performance polymeric materials.^[^ ^88^ ^]^ Bio‐inspired spider silk faces challenges in mass production due to difficulties in replicating natural spinning processes and scaling up. Recent advancements include using microfluidic chips to better mimic natural spinning and achieve higher protein yields. Mussel‐inspired materials have leveraged catechol chemistry for adhesives and hydrogels,^[^ ^89^ ^]^ though challenges remain in replicating the full complexity of natural proteins as well as regarding biocompatibility.

The achievement of multifunctionality in bio‐inspired systems involves the integration of principles from the structure‐function paradigm and the active materiality paradigm (see section 1). The evolution of 3D printing to 4D reflects the evolution from mono‐material printing to producing multifunctional materials using AM. Instead of static 3D‐printed objects, 4D printing enables 3D‐printed structures to change their configuration or function over time in response to external stimuli such as temperature, light, and water.^[^ ^90^ ^]^ The fourth dimension is time.

**Figure 11:**
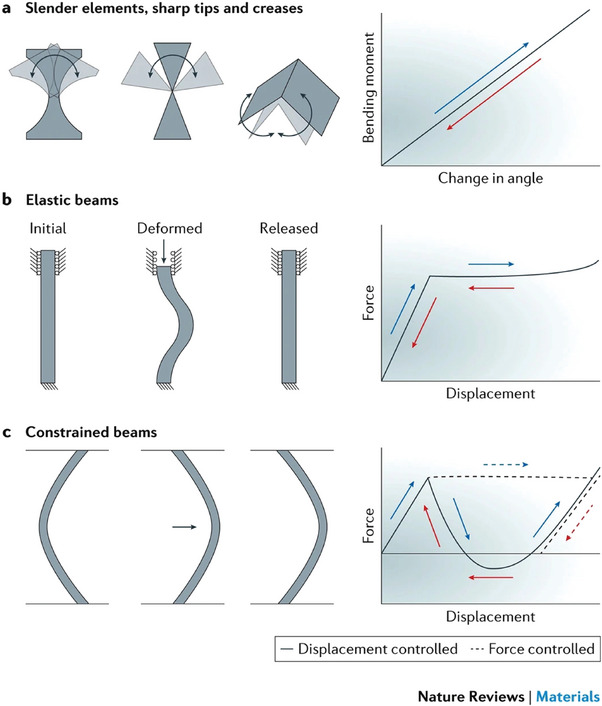
a) Slender elements, sharp tips, and creases localize bending; the bending moment increases monotonically with the angle. b) Elastic beams undergo a buckling instability when axially compressed and fully recover their initial shape when unloaded; this instability provides nonlinear but reversible building blocks for metamaterials. c) Constrained beams can jump to a different equilibrium state through rapid snap‐through buckling. Snapping is often accompanied by bistability, depending on the geometry, amount of confinement, and boundary conditions; for example, experiments in which the external deformations are controlled may result in a different response than in experiments in which the external forces are controlled (solid and dashed arrows, respectively). Hence, under force‐controlled conditions, such elements provide bistable building blocks with hysteretic behavior. Reproduced with permission from Springer‐Nature.^[^ ^17a^ ^]^

AM techniques play a crucial role in enabling the shaping and patterning of so‐called mechanical metamaterials. These materials are not explicitly bio‐inspired but showcase multifunctionality, resembling hierarchically structured biological materials. Metamaterials are designed to achieve certain material properties that exceed those of their bulk counterparts.^[^ ^91^ ^]^ While electromagnetic and optical metamaterials are well‐established, elastic/mechanical metamaterials are still emerging, exhibiting unique functionalities such as shape transformation, unidirectional motion guiding, and varying stiffness or energy dissipation.^[^ ^17a^ ^]^ The building blocks of mechanical metamaterials (**Figure** **11**), known as “meta‐atoms”, deform, rotate, buckle, fold, and snap in response to mechanical forces, with adjacent blocks collectively achieving the desired behaviors.^[^ ^17a^ ^]^ These lightweight, high‐performing structures are spreading rapidly across leading industries, with full‐scale adoption becoming imminent.^[^ ^92^ ^]^ However, optimizing their mechanical properties, especially fatigue resistance, is crucial, as process‐induced defects and microstructural imperfections can significantly impact performance.

Natural hierarchical materials, with their complex microstructures and adaptive abilities, have inspired the creation of hierarchical metamaterials by introducing secondary or higher‐level substructures into primary lattice designs.^[^ ^93^ ^]^ These hierarchical metamaterials can be categorized into three types: those with substructures at the nodes, those with substructures within trusses or walls, and hybrid designs combining both. Such designs significantly improve mechanical properties such as specific stiffness, strength, and energy absorption compared to traditional structures. For instance, hierarchical honeycombs and lattice structures have demonstrated increased stiffness, adjustable Poisson's ratios, and enhanced energy absorption capabilities.

The potential to combine elastic, plastic, and viscous materials can lead to novel mechanical meta‐behaviors, and the fusion of materials with specific functionalities may create hybrid classes of metamaterials such as opto‐mechanical, thermo‐mechanical, or electro‐mechanical materials. This demonstrates the interconnectedness of attaining multifunctionality through structure‐function relations and the material activity concept.

### 3.2 Modularity, Controlled Disassembly, and Reuse of Structures in Bio‐inspired Materials

Creating non‐permanent assemblies, such as reversible bonds, adhesion layers, and modular structures, opens up avenues for enhancing sustainability through controlled disintegration and promoting the reusability of materials, thus fostering a circular materials economy as well as the longevity of material systems.

At the molecular level of biological materials, adhesion layers often represent what are known as sacrificial bonds, which contribute to the unique blend of high strength and toughness. Furthermore, such layers allow for the potential disintegration of the materials if the adhesion mechanisms are reversible due to noncovalent interactions (see section 2.5). Researchers have introduced both noncovalent and covalent sacrificial bonds into synthetic materials such as hydrogels and elastomers, markedly enhancing strength and fracture toughness.^[^ ^94^ ^]^ However, they do not yet enable controlled disassembly. Mimicking molecular structures and bonds relevant in silk adhesion, such as van der Waals forces, hydrogen bonding, and electrostatic interactions, could lead to tailored adhesion in synthetic systems. Surface characteristics such as wettability and capillarity, as found in silk, can be adopted in synthetic materials to influence their adherence and promote interactions with other surfaces. This will allow chemical engineers to design synthetic materials with tailored adhesion capabilities as well as designing the adhesion layers between building blocks. Silk‐inspired materials with adhesive characteristics offer diverse applications including anti‐adhesion coatings, adhesive mucus, and adhesive tape membranes.^[^ ^95^ ^]^ Supramolecular chemistry allows material synthesis using materials that are dominated by noncovalent interactions, such as hydrogen bonding, host–guest interactions, and electrostatic interactions.^[^ ^96^ ^]^ These interactions are reversible and sensitive to external stimuli. Supramolecular materials are highly ordered at the nanometer scale and therefore readily tunable at the molecular level. In principle, information programmed into the molecular‐scale building blocks can be translated through the nano‐ and micro‐scales up to the macroscopic level.^[^ ^97^ ^]^

At higher levels, another example is modular structures with soft and hard elements that offer both robustness against failure and repairability through segmental design. In synthetic materials, such tessellated structures would not only allow the enhancement of controlled disintegration but also the tuning of mechanical properties by subdividing the surface or volume of a relatively hard material into tiles and connecting them via softer layers.^[^ ^62^ ^]^ Certain tessellated geometries involving soft/flexible (polymeric) and hard/stiff (ceramic) elements exhibit intriguing functionalities such as signal filtering and “stretch and catch” responses. In this context, a composite material composed of stiff blocks linked by soft interlayers displays higher compliance under tension compared to compression. Consequently, the composite maintains its stiffness in compression. Such bio‐inspired modular material designs integrating soft/extensible and hard/stiff elements could thus become a valuable tool for achieving customized properties as well as improved sustainability characteristics.

**Figure 12:**
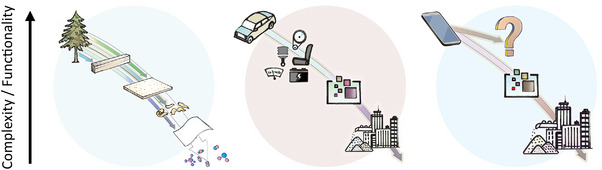
(Left) The reuse of wood after its primary purpose or recycling structural wooden elements of higher hierarchical levels plays a pivotal role in minimizing waste and fostering sustainability. (Center) Synthetic processes are usually bottom‐up approaches in which materials are made from basic elements and broken down again into these basic elements for reuse. (Right) Technical cycles could be adapted in such a way that higher order structures and components could be reused without going back to the elemental level. The reuse of components and materials at higher hierarchical levels in both human practices and natural systems contributes to more efficient resource utilization. Figure by Konrad Eyferth.

The concept of reusing material structures at higher hierarchical levels represents a sustainable strategy aiming to enhance circularity as well as reduce the energy consumption that would otherwise be required for the disassembly of materials into their fundamental constituents, followed by the subsequent reconstruction of similar material structures from scratch. Nature offers a compelling illustration of this “hierarchical reuse” in the context of wood, where a tree can be transformed into various forms such as boards, planks, wood particle boards, or paper, with some of these processes involving enzymatic degradation mechanisms (**Figure** **12**a). In natural systems, growth predominantly follows a bottom‐up approach, where structures develop incrementally. Conversely, synthetic materials, such as polymers, are typically derived from petroleum through catalytic processes and subsequent processing at higher length scales. Figure 12 portrays a vertical axis defining distinct hierarchical levels, each corresponding to varying degrees of intricacy and functional sophistication. This representation emphasizes that the lateral transference of material structures to other products – without disassembling them into lower‐level hierarchical structures – has the potential to augment circularity capabilities and foster energy conservation.

Our proposition is to establish the practice of reusing higher‐level structures in engineered materials, enabling the direct transfer of material functionality at corresponding length scales to new products. For example, this is to some extent already taking place within the automotive industry, where numerous components, including wheel rims, seats, mirrors, displays, electronic components, microchips, and others, can potentially be reused in the manufacturing or repair process (Figure 12b). This prompts us to consider the feasibility of implementing similar strategies in products such as smartphones, envisioning not only their eventual disassembly into electronic waste but also creating their initial design with the intention of reusing certain components in other contexts (Figure 12c). The adoption of this “hierarchical reuse” principle has the potential to significantly enhance resource utilization efficiency, thereby contributing to more sustainable and environmentally responsible practices in material and product design.

Wood, collagenous tissues, and chitin are representative examples of materials that make up substantial quantities of the “waste” that industries generate, even though this waste material has further unexploited potential for more sustainable utilization. Nevertheless, the effective recycling and repurposing of wood must confront various challenges, including issues arising from wood treatments, the presence of synthetic adhesives in wood‐based products, and the presence of contaminants.^[^ ^98^ ^]^ The proper sorting and processing of waste wood can enhance recycling. Although challenges persist, addressing aspects of adhesive‐free wood‐joining techniques and bio‐based glue alternatives will contribute to more sustainable wood product management and utilization.

Traditionally, the industrial utilization of wood has predominantly focused on obtaining segments of tree stems possessing exceptional mechanical properties and relatively consistent material characteristics. However, not all components of trees are directed toward “high‐value applications” due to their inherent geometrical limitations or inferior material attributes.^[^ ^99^ ^]^ For example, branches and tree bark are mainly seen as low‐value or even waste material. However, bark and other “waste products” from trees can be utilized for different applications, especially if one knows the specific properties of these fibrous products. For example, they can be used for textiles (with a leather‐like appearance being achieved through rather simple treatments), temporary huts, or furniture.^[^ ^99^ ^]^

A review on plant‐biomass‐based hybrid materials, incorporating nanoparticles such as nanocellulose and lignin, has highlighted their potential in the development of lightweight, functional, and sustainable materials.^[^ ^100^ ^]^ For such future materials solutions, potentially contributing to the controlled disassembly and reuse of structures, it is particularly important to take into account surface characteristics and the interactions of the plant‐based nanomaterials with aqueous and nonpolar media, polymers, proteins, and cells. For nanocellulose, significant advances have recently been made in understanding polymer adsorption and optimizing hydrogels for biomedical applications.^[^ ^100^ ^]^ However, challenges remain in enhancing interactions with living cells and scaling up production.

Collagen‐rich animal waste is nowadays considered biomass, and it can be derived from a variety of animal sources, including bovine, porcine, avian, and piscine tissue and fish skin waste.^[^ ^101^ ^]^ The processing of such “waste” to create collagen‐based materials has the potential to provide sustainable multifunctional materials such as fibers, films, gels, membranes, and scaffolds. For biomedical applications, such as collagenous scaffolds, higher‐order structures of collagen are being reused. Therefore, a complete denaturation of the collagen should be avoided in order to keep the integrity of the desired structural levels. However, collagen processing often involves the destruction of cross‐linkages between polypeptide chains of collagen along with some breakage of polypeptide bonds.^[^ ^102^ ^]^ Thus, to promote the sustainable use of collagen products, a compromise needs to be found between the necessary disintegration of higher‐order collagen structures during the processing steps and a later recovery of the bonds, e.g., a reintroduction of intermolecular, covalent cross‐links between collagen fibrils.

Chitin is a natural polymer and, together with its derivative chitosan, has various applications in agriculture, biomedicine, and the food industry. It can be extracted in large amounts from natural sources but often needs to be processed to optimize its structural and chemical parameters, such as molecular weight distribution and the degree of acetylation, for further usage.^[^ ^103^ ^]^ The raw material for chitin production originates from insects and microorganisms, but the main sources are the cuticles of various crustaceans, primarily crabs and shrimp. To achieve high purity, proteins, minerals, lipids, and pigments have to be removed by chemical (via acids and bases) or biological (via microorganisms) methods. The removal of proteins and minerals has detrimental effects on the molecular weight and degree of acetylation. However, processing methods aim to minimize the degradation of the chitin while simultaneously achieving sufficient purity levels for specific applications. One of the drawbacks of chitin‐based products is that they are currently significantly more expensive than traditional synthetic plastic‐based products.^[^ ^104^ ^]^

In summary, integrating modularity, controlled disassembly, and the reuse of material structures in products is a crucial step toward materials sustainability.

### 3.3 Bio‐Inspired Material Examples for Failure Prevention or Post‐failure Recovery

As a complement to section 2.6, this section serves as an exposition of bio‐inspired material concepts that are specifically designed to address two fundamental aspects of material performance: i) the prevention of failure and ii) the restoration of material functionality in the event of failure, both contributing to longevity of materials.

Some synthetic materials incorporate structural redundancy inspired by biological systems, ensuring functionality even when parts are damaged. Honeycomb materials, used in aerospace and engineering, have an exceptional strength‐to‐weight ratio (see section 2.4). They dissipate stress through interconnected cells, maintaining structural integrity despite damage. Lightweight sandwich structures are essential for sustainable, high‐performance designs, with the core enhancing rigidity and controlling failure mechanisms.^[^ ^105^ ^]^ In addition, some of the materials mentioned in the previous section that have noncovalent bonding between building blocks can be considered as failure‐resistant as they exhibit higher toughness and may show higher fatigue tolerance than more rigid materials.

Another example of a very special type of human‐driven remodeling—and thus a preventive strategy—can be found in Japan, where there are thousands of Buddhist temples with a long history. The main temple hall is the central architectural structure, and it is usually made from wood and maintained by artisans such as temple and shrine carpenters.^[^ ^106^ ^]^ The aging of wooden structures involves the deterioration of structural elements, which are continuously replaced, so that the materials is renewed without any disruption of the functionality of the building. The Buddhist temples of Nara, Japan, are described as follows by the Japanese Agency for Cultural Affairs: “Wooden structures require careful routine maintenance for conservation from the time they are constructed. For the wooden Buddhist structures in the Horyu‐ji area, the temple organizations have continued appropriate conservation activities for 1300 years, repairing and caring for these buildings originally with the support of the Emperor and later with the support of the central government”. (From https://bunka.nii.ac.jp/suisensyo/horyuji).

Bio‐inspired self‐healing polymers mimic the regenerative abilities found in nature. These materials have the capability to autonomously repair small defects or damage that may occur during their lifespan. Three main conceptual approaches for self‐healing have been demonstrated: capsule‐based, vascular, and intrinsic healing systems.^[^ ^107^ ^]^ For example, microcapsules containing healing agents can be incorporated into a polymer matrix and, when a crack or damage occurs, the capsules rupture, releasing the healing agents to fill and repair the defect, thus restoring the material's integrity. Self‐healing can be autonomous or may require external energy.

Fiber‐reinforced composites are generally inspired by the hierarchical structure of natural materials, but self‐healing mechanisms can also be incorporated. The interconnected network of fibers in such composites provides structural redundancy, distributing stress and load across multiple paths. This redundancy allows the material to maintain its integrity even when some fibers are damaged, making it highly resistant to fractures. Despite extensive testing of self‐healing systems in fiber‐reinforced polymers, almost none have been commercially applied. There are various factors contributing to this lack of commercialization. A review by Cohades et al. summarizes the following advantages and disadvantages:^[^ ^108^ ^]^ Microcapsules offer autonomous activation and eliminate the need for stoichiometric mixing but increase manufacturing complexity and can degrade mechanical performance. Vascules support large healing volumes and extensive reusability but significantly increase manufacturing complexity and can degrade mechanical performance. Intrinsic systems are easy to manufacture and provide extensive reusability but have very small healing volumes and can severely degrade baseline performance if not properly managed.

Self‐healing injectable hydrogels, which can fluidize under shear stress and recover their original properties, offer significant advantages for tissue regeneration, including minimally invasive administration and patient‐specific interventions.^[^ ^109^ ^]^ Such hydrogels support tissue regeneration by providing mechanical support and the controlled delivery of cells or therapeutics.

## 4 Conclusion and Outlook

In summary, this article has systematically explored the domain of materials and their sustainability through a bio‐inspired lens, yielding new insights and paradigms that reshape our comprehension of material usage. Our journey began with the recognition of materials as foundational elements in technological advancement, serving a wide array of purposes from structural applications to healthcare solutions. The accessibility of materials, whether scarce or abundant, necessitates a spectrum of processes, including extraction, purification, synthesis, and processing. All these processes represent relevant targets for the enhancement of materials sustainability. A blueprint for the efficient recycling and reuse of materials can generally be observed in natural ecosystems. To achieve higher sustainability in synthetic materials, it is crucial to focus on material processing. Functionality in materials derives not just from their chemical composition but also from their structure, which can be modified through processing to fit various applications. This approach aligns with the natural world, where hierarchical structuring during growth imparts functional diversity and emphasizes the importance of recycling and transforming waste materials to serve new applications.

In the field of anthropology, the concept of “materials ecology” explores the dynamic connections between materials and their surroundings, including the social and cultural aspects of these connections. It examines the entire life cycle of materials, from extraction to use and disposal, considering environmental, economic, and social effects. Shifting focus from static material objects to dynamic processes within a larger ecological system, materials are viewed not just as resources but as constantly changing entities. Materials are seen as processes of making, emphasizing dynamic relationships with the natural and cultural world. We have embraced this perspective from a bio‐inspired engineering angle.

Computational tools, including molecular modeling and machine learning, play a key role in designing future sustainable materials by (i) drawing inspiration from nature's strategies and (ii) enabling precise property predictions and simulating complex interactions.^[^ ^110^ ^]^ Recent advancements in mesoscale modeling and generative methods further enhance our ability to develop novel materials from biomass and other sustainable sources. To fully harness these tools, it is essential to establish quantifiable sustainability metrics and continue exploring how computational methods can drive progress in sustainable material design. Bio‐ or life‐inspired materials offer attractive functionalities, but sustainability and resource efficiency are nowadays even stronger drivers for advancing biological manufacturing.^[^ ^20d^ ^]^ As production scales, the shift away from fossil carbon and toward energy efficiency becomes crucial. The integration of biological organisms into material development, enabling features such as self‐repair and autonomous functions, is a global trend that is expected to significantly impact future material science, particularly in the context of environmental and resource sustainability.

We explored how various structural features, functions, and concepts in biological materials can contribute to enhancing sustainability in the realm of materials science. By examining nature's creations, we observed the implementation of concepts such as mono‐materiality and multifunctionality in order to streamline material variety, as well as seeing how responsivity, adaptivity, modularity, and cellularity can simplify material assembly and disassembly. Additionally, we delved into the strategies of reusing materials and structures, defect tolerance, maintenance, remodeling, and healing, all aimed at extending the lifespan of products. We took the foundational sustainability principles – circularity, longevity, and parsimony – and transformed them into dynamic bio‐inspired paradigms. This led us to revisit the classic perspective of material science as being mainly concerned with the relationship between structure and function and extend it by introducing the concept of “active materiality”. This view opened up new strategies for the production of complex materials that are able to respond or adapt to external stimuli. Furthermore, we provided concrete examples from the fields of engineering and technology, illustrating how bio‐inspired strategies have been or are on the verge of being integrated to enhance the sustainability of materials’ life cycles. We have tried to challenge the conventional portrayal of materials as passive components, and this prompted a reconsideration of the applicability of conservation laws governing mass and energy to material functionality. Notably, the emergence of metamaterials and architectured materials exemplifies the fact that the properties of a material do not solely derive from its composition but are also influenced by geometric arrangements, as seen in the creative strategies found in biological materials.

The exploration of bio‐inspired materials concepts culminated in the recognition of the indispensability of a multifaceted approach to material sustainability, extending beyond the conventional building‐block view of materials. Furthermore, a shift from a circular to a network conception could form the basis for a crucial evolution in materials sustainability. While the division of labor in manufacturing processes has undeniably propelled economic efficiency, it has also contributed to the stabilization of material cycles, hindering recycling and reuse strategies. Replacing a linear cycle in production processes with a network‐based approach may in future enable new design concepts, fostering sustainability across the entire value chain.

In conclusion, this systematic journey has illuminated the path toward addressing material sustainability from a holistic, interdisciplinary perspective, transcending disciplinary confines. It underscores the necessity of breaking down barriers and fostering collaboration between various fields of expertise. This transformative shift, while undoubtedly challenging, holds the key to developing a more sustainable, resilient, and dynamic materials economy that harmonizes with the intricate flow of nature's material cycles.

## Conflict of Interest

The authors declare no conflict of interest.
